# Supplementary figures and images for: Real-world utilization of SARS-CoV-2 serological testing in RNA positive patients across the United States
Source: PLoS One. 2023 Feb 10;18(2):e0281365. doi: 10.1371/journal.pone.0281365 (PMC9916659; doi:10.1371/journal.pone.0281365)

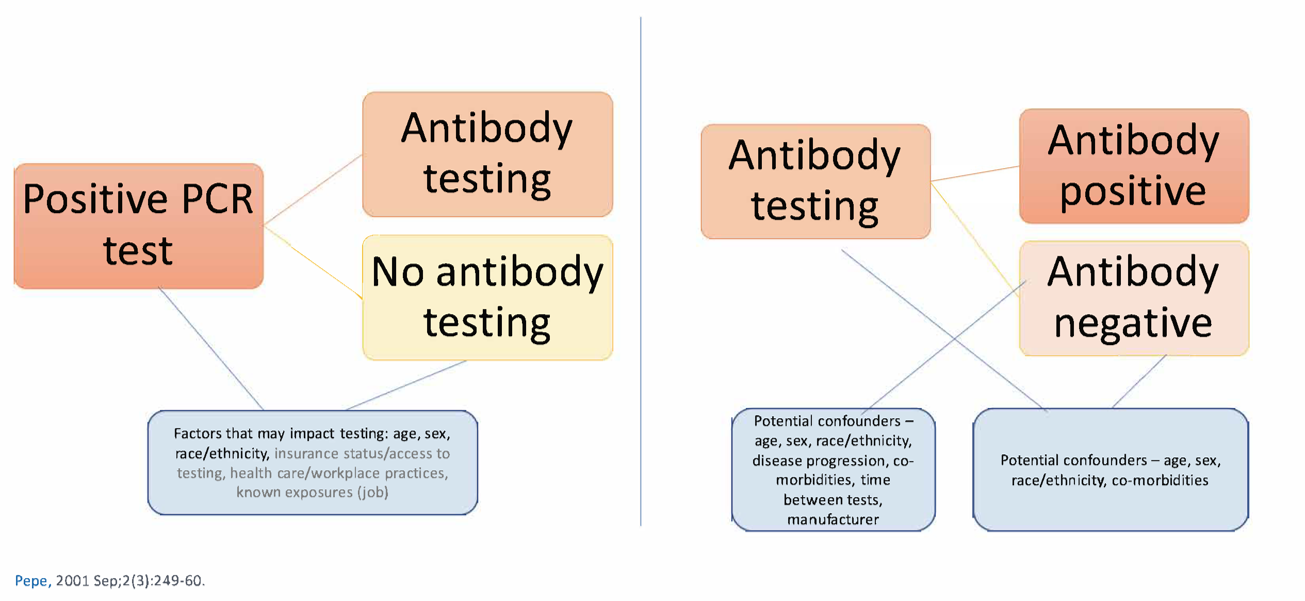

Supplement: S1 Fig — (TIF) [file pone.0281365.s003.tif]
