# Supplementary material for: Real-world utilization of SARS-CoV-2 serological testing in RNA positive patients across the United States
Source: PLoS One. 2023 Feb 10;18(2):e0281365. doi: 10.1371/journal.pone.0281365 (PMC9916659; doi:10.1371/journal.pone.0281365)
Supplement: Attachment — Submitted filename: Edgeworth PLOS 21 30196.docx [file pone.0281365.s004.docx]

This is an impressive dataset collected from multiple systems addressing principles of inter-operability, population coverage and linkage of laboratory results with detailed clinical metadata to address policy or public health needs. The main concern is whether the objectives set have made sufficient progress to justify publication at this point. It also would benefit from more detailed explanation of the research questions behind the objectives set (presumably by the FDA) to support presentation of the progress made in the form of a research publication, rather than alternatively as a published interim project progress report. It also seems aligned to the requirements of the US approach to FDA diagnostics approvals, implementation and guidance. This makes it a bit hard to clearly see the specific research questions as opposed to providing data to support decision making within the US diagnostics regulatory framework.

The comments below expand on these concerns which are raised from perspective of a non-US infectious diseases clinician involved in SARS-CoV-2 testing including serological tests. Consequently some concerns may be easily addressed or reflect misunderstanding due to lack of familiarity with the US setting and how this work fits in.

Abstract - conclusion: how exactly does this methodological approach actually address the question of determining performance data or linking with manufacturer and clinical data.

It does not seem appropriate to say the need for more efficient testing strategies without presenting the actual strategies and rationale for testing in these different settings at that time. Given the dynamic nature of this disease there is potential for findings of a study like this to not be relevant by the time a follow up study is done or recommendations are made for the future.

For example, in the study period serology testing was sometimes performed to help with diagnosis of what was a new condition. Antibodies appear around day 10 and so without vaccination or potential for prior infection, it was not inappropriate to perform serology to help with diagnosis. Duration of symptoms was an important clinical measure not collected in this study.

In addition, patients now get serology testing on admission to determine which SARS-CoV-2 RNA-PCT positive patients are antibody negative and might require Regeneron or similar

Finally, now that vaccination is widespread serology testing is used more to assess immunity (not prevalence/incidence of disease) or as a universal hospital admission test to inform infection control decisions (ie antibodies +/- history of vaccination = protected and less likely to acquire SARS-CoV-2).

Line 69 serology is not only done to determine prevalence estimates

Line 73 Please explain for those not familiar with US regulatory processes what the relevance of 510(k) is and what the link is between approvals and this collection of real world evidence

Line 81 -83. Interoperability and linkage of tests with metadata from EHR impacts on all of medicine but what are the advantages of this approach here for determining test performance and linking with clinical symptoms. Please explain the benefits compared with studies conducted at representative institutions / different clinical settings where local laboratory and clinical data can be collected alongside guidelines and rationale. Realise the national approach will get there but it is a long and complex road to get to the granularity required to meet stated objectives.

Line 93. The FDA asked for this consortium to be established with specific purpose. The consortium has a broad range of experts and links diverse providers. Line 100 sets 3 different objectives. How does the meeting of those objectives link into a research question and publication?

Line 109: How did these organisations came together and are representative of the organisations involved in serology. They presumably were not randomly selected. The authors do say heavy representation from certain states

Line 126. March to September was very early on in the pandemic. First wave. Diagnostics and guidelines have improved significantly. All tested parameters are very different now, hence making generalisability comments, even for the US, seems tenuous for today.

From line 128: it is hard to understand the locations and factors underpinning access to diagnostic tests from outside the US system to interpret results and assess potential biases and confounders

Line 251; Duration of symptoms is an important measure in determining whether to perform serology testing alongside PCR testing at time of first presentation

Line 278: What is is the clinical purpose of doing routine follow up serology testing in a PCR confirmed SARS-CoV-2 patient.

Line 317. Don’t agree with this statement. As mentioned above there have been many different reasons for doing antibody testing. Without incorporating perspectives of what is happening on the ground as things change fast, it is inappropriate to link with unreferenced and likely now updated “clinical guidance”

Line 327: data of PCR test is not a good proxy for date of symptom onset. PCR testing in many studies is reported to be done between 5 and 15 days post onset of symptoms when patients present to healthcare. Accept this may be different in the US
