# Supplementary material for: Real-world utilization of SARS-CoV-2 serological testing in RNA positive patients across the United States
Source: PLoS One. 2023 Feb 10;18(2):e0281365. doi: 10.1371/journal.pone.0281365 (PMC9916659; doi:10.1371/journal.pone.0281365)
Supplement: Attachment — Submitted filename: PONE D21 30196.docx [file pone.0281365.s006.docx]

PONE D21 30196

I am happy with this paper because the authors have responded to comments and concerns well, particularly by adding explanations, explaining limitations better and limiting their conclusions to what is justified for this real-world observational study. I can see they have also responded appropriately to the comments from other reviewers.
