# Supplementary material for: Real-world utilization of SARS-CoV-2 serological testing in RNA positive patients across the United States
Source: PLoS One. 2023 Feb 10;18(2):e0281365. doi: 10.1371/journal.pone.0281365 (PMC9916659; doi:10.1371/journal.pone.0281365)
Supplement: Attachment — Submitted filename: Response to Reviewers-Response 2.docx [file pone.0281365.s007.docx]

Dear Dr. Liu,

We thank you and the reviewers for your thoughtful comments on our manuscript. We appreciate the opportunity to respond and believe the revisions have improved the manuscript. Below, please find a table with the summary of reviewer comments and our responses. We look forward to hearing from you.

Regards,

Carla Rodriguez-Watson, PhD, MPH

Director of Research

Reagan-Udall Foundation for the FDA

| **Reviewer Comments** | **Response** |
| --- | --- |
| **Reviewer 1:** | |
| 1. Tables pages 10 - 15: Thank you for adding the total column to the table. However, the column shows a total for both "Yes" and "No" for serological testing status which adds little information. This reviewer feels it will be better to show a Total column for "Yes" and a total column for "No" in order to compare the broad outcomes of serological testing in each group. | Thank you for your comment. The comment was unclear to us. If the request is to combine all the Yes’s across all the datasets (i.e. 2191+14059+2170+2808+2137+12441), as well as No’s, that would not be appropriate because the data are not homogenous and derived from different data source types (e.g., EHRs, claims). Given the unique features of each data source (e.g., variable data availability, completeness, and characterization), this study consisted of parallel analyses (i.e., implementation of a common protocol and analysis plan in separate unique datasets) with none of the analyses representing a combined dataset. |
| 2. Table, page 23: "Any Chargemaster or Medical Claim". Please clarify in the footnotes what "Chargemaster" is. As a non-US reader/reviewer, I am not sure of the relevance of this. | Thank you for your comment. In the datasets, any chargemaster or medical claim refers to a comprehensive list of a hospital’s billable products, procedures, and services. We have added this description to the legend of Table 3. Please see lines 291-293. |
| **Reviewer 2:** | |
| 1. Has the statistical analysis been performed appropriately and rigorously? | Thank you for the question. Yes, we conducted the statistical analysis appropriately and rigorously. Our objectives for this study were to 1) understand the current state of data interoperability across instrument, laboratory, and clinical data; 2) describe serological testing by demographic, environmental characteristics (e.g., geographic location), baseline clinical presentation, key comorbidities (e.g., diabetes and cardiovascular disease), and bacterial/viral co-infections (e.g., influenza), and 3) assess the timing of serology testing relative to molecular testing date by the characteristics listed above. Given these objectives, we performed a number of statistical analyses. Descriptive analyses were performed on the covariates of interest separately by each contributing data partner in accordance with a common analytic plan. Additionally, we calculated the median and interquartile range (IQR) for the number of days between RNA and the first test. Separately, we included all serology and RNA tests after the index date to describe the median and IQR for the number of molecular and serological tests conducted after the index date. A complete description of the statistical analysis that was performed can be found in lines 178-190. |
